# Being a Patient Representative Is More Work Than Being a Patient Advocate—Done Right, It Is Also Likely to Be More Effective 

**DOI:** 10.3390/curroncol28020099

**Published:** 2021-02-25

**Authors:** Richard Wassersug

**Affiliations:** Department of Cellular and Physiological Sciences, University of British Columbia, Vancouver, BC V6T 1Z4, Canada; richard.wassersug@ubc.ca; Tel.: +1-(604)-347-5601

**Keywords:** patient advocacy, patient representation, oncology

## Abstract

For a patient to be effective as a “patient representative” within a health-related organization, work and more than just accepting an honorific title is required. I argue that for a patient to be most effective as a patient representative requires different types of background knowledge and commitment than being a “patient advocate”. Patients need to be cautious about how, when, and where they take on an official role of either an “advocate” or “representative”, if they truly want to be a positive influence on health outcomes.

This essay is about a couple of common labels applied to patient activists—individuals devoted to improving healthcare outcomes for others well beyond their own healthcare needs. These labels are catch phrases meant as platitudes, but they also pigeonhole patients. These are terms that have subtle but significant differences that can influence the success of an activist’s mission, whatever it might be.

By way of background, I am both a prostate cancer patient and a research scientist who studies ways to improve the quality of life of cancer patients. I routinely co-author academic papers on the psychological burden brought on by cancer treatments and I have collaborated extensively in this research with physicians, although I am not one myself.

Because of my status as a patient, I ended up as a patient representative for an organization that makes recommendations on how funds raised for cancer research in Canada should be distributed. My formal title is “Patient Representative”, but I have occasionally been addressed by colleagues as a patient advocate.

The word “advocate” comes from the Latin “ad” plus “voca” meaning to “speak to or for” a cause or a person. In the clinical setting, the first and foremost advocate for the patient is their physician. This is because practicing medicine begins with acquiring first-hand knowledge about patients’ needs and concerns. If patients need others to speak on their behalf, however, a non-clinician advocate may be warranted. I acknowledge that there are situations where patients truly need advocacy beyond what their healthcare providers can provide. Unfortunately, implicit in that situation is that some patient needs are not being met by their healthcare providers. As a result, there is an unavoidable confrontational aspect to being a patient advocate. 

Patient representatives have different responsibilities than patient advocates and hopefully they can be effective without being, or being perceived as being, adversarial. I am just one of 15 patient representatives helping a national organization to evaluate cancer research proposals. We are dispersed among different cancer types. None of us is an MD, but nor are we patients picked randomly off the street. I cannot speak for all the patient reps, but the vast majority take the title of “Patient Representative” seriously and recognize a responsibility to other patients afflicted with our diseases, both now and in the future, whether we know them personally or not. To meet this responsibility, we spend a significant amount time trying to stay up to date on advances in cancer treatments. We also work hard to be well-informed about the concerns of patients with these conditions. In my view, a patient representative has an undefined yet incarnate constituency. 

It is hard to be effective as an advocate if one does not personally know one or more of the individuals one is fighting for. By contrast, to be an effective representative, one does not necessarily need to know the unique angst of individuals in the community. Instead, one needs to be familiar with the dominant yet evolving concerns of the community overall.

Being imprecisely labeled as a patient advocate may seem trivial, but it could mislead my clinical colleagues to think I have an agenda that includes some criticism of the care provided by physicians. That is neither true nor relevant in those working sessions where we collectively struggle to understand and evaluate proposed research projects.

In these meetings, the common concern is the quality of the science in the proposals before us. I am not there to advocate on behalf of patients of the panel members, or any patients for that matter. The MDs on the panel are among the best physicians and medical researchers in the country, or else they would not be in the room. As such, their patients do not need advocacy from a non-clinician, least of all during our panel meetings.

In other settings, patient representatives may have less responsibilities and be offered a seat at the table to affirm an organization’s transparency. Patient representation may then be more akin to tokenism and the title “Patient Representative” honorific, requiring no major time commitment or special skill set.

Healthcare organizations increasingly find it politically expedient to park patients on panels to demonstrate their commitment to the current catchphrase “patients as partners”. Patients in those settings are often just observers, not decision makers. Unfortunately, I have met many activist patients who have not given much thought to who they represent and their responsibility to other patients. Simply holding the title “Patient Representative” does not imbue one with unique insight on healthcare. It can thus be embarrassing to witness a tokenized patient representative make proclamations to seasoned physicians about “the” patient experience. 

Patient representatives need to be cognizant of the fact that the physician’s profession is built upon their experience learning of and responding to the critical needs of their patients. If patient representatives are going to be helpful in advancing discussions with healthcare providers, they need to have novel insights drawn from a large sample of patients beyond what physicians come across in their daily practice. Personal opinions should hold little importance in this setting. In sum, being an effective patient representative requires significant time, dedication, and effort. 

Returning to the term advocate, organizations in the cancer world that promote this label also typically have a rule about not crossing the line and having patient advocates provide medical advice. But could there be exceptions to this? I believe there may be one for scientists, like myself, who are not MDs, but professionally study disease-specific treatments and their side-effects. I certainly do not offer medical opinions unless asked. But I do get asked and I preface anything I say with the boilerplate disclaimers that I have no clinical credentials whatsoever. 

So how should I respond when my physician colleagues ask me for my opinion on prostate cancer treatments? They want a well-informed, evidence-based opinion. So, what do I do? 

I treat them with the same respect that they treat me when they see me in the clinic. I give them the best advice I can take from the medical literature and encourage them to get back to me about their outcomes. 

I will close with one of the more ironic opening lines from one of my close friends, who is also a prostate cancer patient and a physician. He recently started a conversation with me with, “Richard, I got some test results back and was hoping I could talk to you about them. I’m not asking your medical advice, but…” I cut him off. I said “Yes, you are asking me for my medical advice. So how can I help?” 

He broke out laughing and thanked me for being there. 

If I trust my physicians to advocate on my behalf, the honorable thing to do is to reciprocate.

